# AI's ability to interpret unlabeled anatomy images and supplement educational research as an AI rater

**DOI:** 10.1002/ase.70074

**Published:** 2025-07-11

**Authors:** Lord J. Hyeamang, Tejas C. Sekhar, Emily Rush, Amy C. Beresheim, Colleen M. Cheverko, William S. Brooks, Abbey C. M. Breckling, M. Nazmul Karim, Christopher Ferrigno, Adam B. Wilson

**Affiliations:** ^1^ Rush Medical College Rush University Chicago Illinois USA; ^2^ Academic Affairs Rush University Chicago Illinois USA; ^3^ Department of Anatomy and Cell Biology, Rush Medical College Rush University Chicago Illinois USA; ^4^ Department of Medical Education, Marnix E. Heersink School of Medicine University of Alabama at Birmingham Birmingham Alabama USA; ^5^ Department of Anatomy and Cell Biology, College of Medicine University of Illinois at Chicago Chicago Illinois USA; ^6^ School of Public Health and Preventive Medicine Monash University Melbourne Victoria Australia

**Keywords:** AI in research, anatomy images, ChatGPT, Claude, generative artificial intelligence

## Abstract

Evidence suggests custom chatbots are superior to commercial generative artificial intelligence (GenAI) systems for text‐based anatomy content inquiries. This study evaluates ChatGPT‐4o's and Claude 3.5 Sonnet's capabilities to interpret unlabeled anatomical images. Secondarily, ChatGPT o1‐preview was evaluated as an AI rater to grade AI‐generated outputs using a rubric and was compared against human raters. Anatomical images (five musculoskeletal, five thoracic) representing diverse image‐based media (e.g., illustrations, photographs, MRI) were annotated with identification markers (e.g., arrows, circles) and uploaded to each GenAI system for interpretation. Forty‐five prompts (i.e., 15 first‐order, 15 second‐order, and 15 third‐order questions) with associated images were submitted to both GenAI systems across two timepoints. Responses were graded by anatomy experts for factual accuracy and superfluity (the presence of excessive wording) on a three‐point Likert scale. ChatGPT o1‐preview was tested for agreement against human anatomy experts to determine its usefulness as an AI rater. Statistical analyses included inter‐rater agreement, hierarchical linear modeling, and test–retest reliability. ChatGPT‐4o's factual accuracy score across 45 outputs was 68.0% compared to Claude 3.5 Sonnet's score of 61.5% (*p* = 0.319). As an AI rater, ChatGPT o1‐preview showed moderate to substantial agreement with human raters (Cohen's kappa = 0.545–0.755) for evaluating factual accuracy according to a rubric of textbook answers. Further improvements and evaluations are needed before commercial GenAI systems can be used as credible student resources in anatomy education. Similarly, ChatGPT o1‐preview demonstrates promise as an AI assistant for educational research, though further investigation is warranted.

## INTRODUCTION

Recent developments in generative artificial intelligence (GenAI) have made it possible to leverage AI integrations for recreational and educational uses. One notable advancement is GenAI's ability to interpret images. For example, existing AI integrations can identify plants and flowers from photographs to provide specific care instructions tailored to the plant's needs. The transferability of this type of application has potential for anatomy education, prompting the question: “How well can GenAI systems analyze and accurately identify unlabeled images of anatomical structures?”

GenAI innovations in medical education have the potential to bring a new level of learning synergy, fostering engagement between students and systems around classically challenging topics such as anatomy, which often involves extensive memorization, synthesis of complex visual information, navigating anatomical variation, and interpreting various medical imaging modalities (e.g., conventional radiographs, CT, MRI, and ultrasound). One area where students may struggle is studying unlabeled anatomical images or models, which can be ineffective without immediate corrective feedback. In this context, GenAI's ability to interpret images and provide explanatory text holds promise as a valuable resource and learning aid for novice anatomy learners,^1^ though its efficacy for this purpose is not well understood.

Within anatomy education, studies have reported on the ability of commercial and customized GenAI systems to i) accurately respond to text‐based prompts, ii) answer USMLE‐style multiple‐choice questions,^2^, ^3^, ^4^, ^5^ iii) generate anatomical images from text descriptions,^6^ iv) annote ultrasound images,^7^ and augment assessment grading.^8^, ^9^, ^10^ To date, study outcomes on GenAI's usefulness in anatomy education have been mixed, and no reports have critically detailed how well GenAI systems interpret and provide accurate feedback on unlabeled anatomy images for learning purposes. Methodologically, the extant anatomy education literature often isolates study designs to a single anatomical region and lacks test–retest analyses to evaluate the repeatability of GenAI's outputs.^2^, ^4^ The present work attempts to overcome these limitations by investigating two anatomical regions and conducting test–retest reliability analyses.

This pilot study examined the capabilities of ChatGPT‐4o (OpenAI 2024 San Francisco, CA., USA) and Claude 3.5 Sonnet (Anthropic 2024 San Francisco, CA., USA) to interpret unlabeled anatomy images and provide accurate and reliable feedback. Secondarily, the effectiveness of OpenAI's ChatGPT o1‐preview model (released 9/12/2024) was evaluated as an AI rater by comparing its rating accuracy to anatomy experts when evaluating text‐based responses to image‐based anatomy questions. This study's outcomes offer educators and students guidance on the usefulness of GenAI systems for interpreting anatomical images and underscore areas warranting further AI development and exploration. Strategically evaluating the newest AI advancements helps establish additional evidence for responsibly and effectively integrating GenAI tools into curricular, assessment, and administrative practices.

## METHODS

The Rush University Institutional Review Board determined that this study did not meet the definition of human subjects research. All images assessed in this study were used with explicit consent and/or were sourced from works licensed under Creative Commons or designated as Public Domain. The licenses included: Creative Commons Attribution‐NonCommercial‐ShareAlike 3.0 and Creative Commons Attribution‐ShareAlike 3.0.

### GenAI model selection

To better understand GenAI's ability to interpret anatomical images and identify various anatomical structures, this study evaluated the two most common commercial GenAI systems capable of image interpretation: ChatGPT‐4o and Claude 3.5 Sonnet. At the time of the study, ChatGPT interpreted images by analyzing the visual data within the picture, identifying key objects, colors, and patterns, and generating a textual description based on its understanding of the image content. It treated images as a form of text input that it then processed and responded to with natural language.^11^ Claude 3.5 Sonnet's proprietary image interpretation process was not well described. As of November 2024, ChatGPT‐4o held the largest market share (59.4%) in the field of AI development.^12^ Claude 3.5 Sonnet was the comparator, given its purported advanced image interpretation capabilities and similar natural language features. At the time of this study, no other publicly available GenAI systems could interpret images.

### Image preparation

Five musculoskeletal and five thoracic anatomy images representing various image‐based media common to anatomy teaching (i.e., photographs, illustrations, radiographs, MRIs, CTs, and ultrasound) were used in this study (Appendix S1). Musculoskeletal and thoracic anatomy were selected as sample anatomical regions to encompass a breadth of identifiable structures across multiple visual media. All musculoskeletal images depicted shoulder joint anatomy to maintain anatomical consistency across the prompts. Each image was edited using Microsoft Paint Windows 11 (Microsoft Corporation, Seattle, WA, USA) or PowerPoint (Microsoft Corporation, Seattle, WA., USA) to superimpose an orange arrow, star, or circle over the area of interest. All gross anatomy images utilized key anatomical views commonly employed in anatomy atlases or clinical contexts. Screenshots of the edited images and text prompts to guide the query were uploaded to each GenAI system for interpretation. Screenshots, rather than original image files, were used to mimic how students would likely interface with GenAI when asking image‐based anatomy questions. Utilizing a screenshot of an image, instead of directly importing an image, also mitigated the potential use of metadata (e.g., file names with the answers), though neither ChatGPT‐4o nor Claude 3.5 Sonnet utilizes metadata from uploaded images to assist with image analysis.

### Prompt development

**FIGURE 1:**
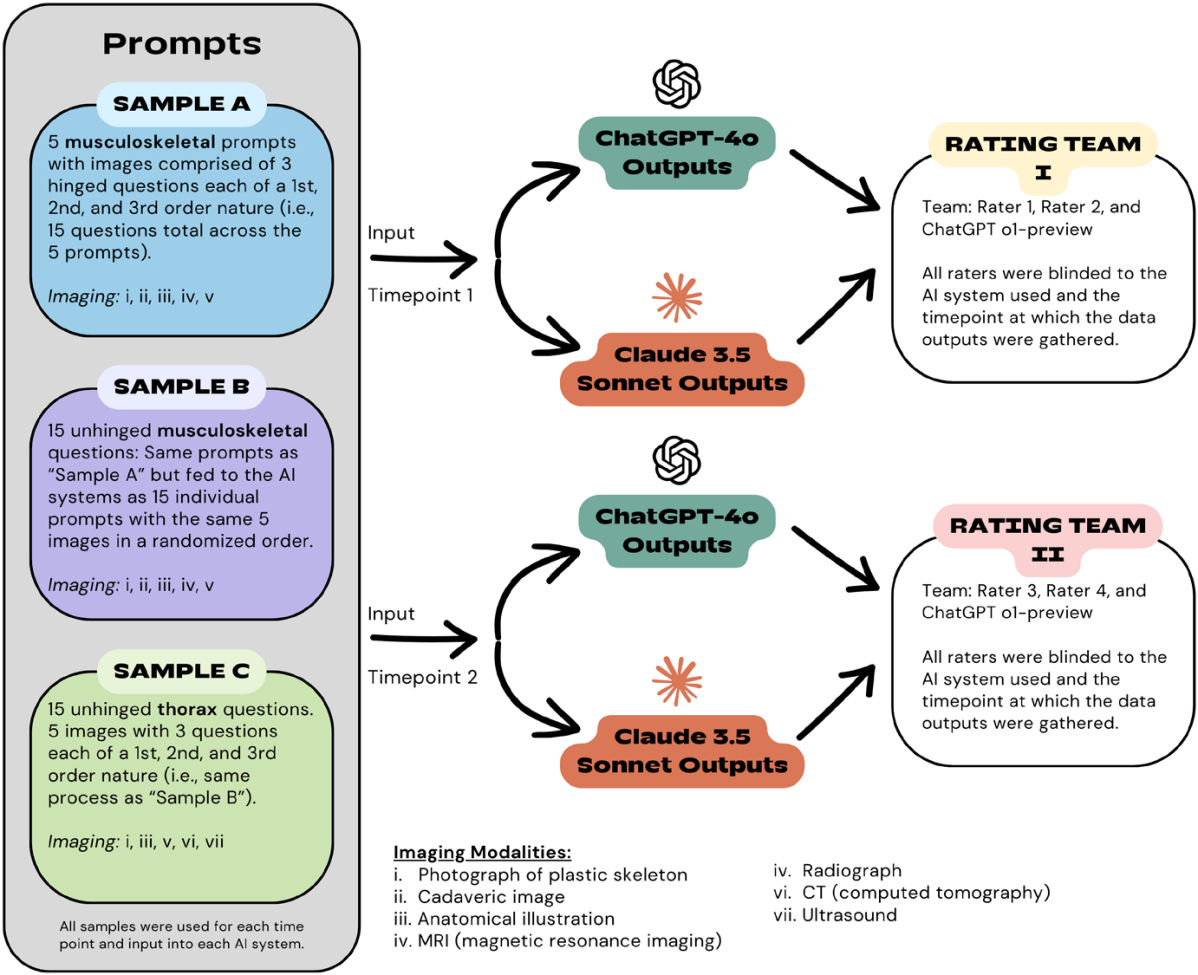
Study design flowchart depicting prompt differences between samples, timepoints for prompt entry, and the structure of the rating teams evaluating the AI outputs.

Two experienced anatomists (ABW and CF) and an AI expert (ER) independently reviewed all images and their respective prompts to ensure their adequacy for the study. Images were deemed adequate if the anatomical structures of interest were clear, had discernible landmarks and relationships to aid in identification, and conformed to a standard key anatomical view. Images with spatially challenging views were excluded. The investigators deliberated and reached a consensus on the final images and prompts. The prompts focused on musculoskeletal and thoracic content and were classified as Samples A, B, and C. The nature of each prompt sample is explained in Figure 1.

The study design also tested hinged versus unhinged item prompts. “Hinged” items represented three orders of questioning (first‐order, second‐order, and third‐order questions) bundled into a single prompt. Unhinged prompts used the exact same questions and unlabeled anatomy images as the hinged prompts, but they were input individually into each GenAI system in a randomized order. This approach aimed to determine whether a GenAI system could achieve more accurate answers by triangulating contextual cues within the three hinged questions. For example, one hinged musculoskeletal prompt with a leader line pointing to the posterior aspect of the deltoid muscle on a human donor image read as follows:

Example Prompt: “You are an anatomy professor helping a first‐year medical student prepare for an anatomy practical examination. Succinctly answer the following questions: (1) What is the anatomical structure marked by the orange star? (2) What motor nerve innervates the structure marked by the orange star? and (3) From which cord of the brachial plexus is the nerve derived that innervates the structure marked by the orange star? Limit your responses to essential information. Return one answer for each question in the same order as the questions were asked.”

For this example prompt, it is plausible that a GenAI system could use the second‐ and third‐order questions to narrow the answer for question 1 to a muscle innervated by a motor nerve branching from the brachial plexus. In doing so, it could rule out the neighboring trapezius muscle and deeper intrinsic back muscles and, thus, plausibly achieve higher accuracy in its answers to hinged questions relative to unhinged items.

### Collecting AI outputs

Three samples of prompts (A‐C) querying anatomical structures across various image‐based media were submitted to both ChatGPT‐4o and Claude 3.5 Sonnet using their online interfaces on September 21–22, 2024. Two investigators (LJH and TCS) submitted each prompt sample across two different timepoints to each GenAI model, typically 24 hours apart (Figure 1). Each prompt was submitted in a new instance of each respective GenAI system to prevent the previous question's context from affecting the subsequent query's output. ChatGPT‐4o's temporary chat feature was utilized, disabling its memory function for storing and learning information from previous conversations to ensure information inputs would not be used to train the model or affect subsequent outputs. Claude 3.5 Sonnet does not support a memory function between chat sessions. Thus, before inputting each prompt, a new chat was created to minimize the influence of context clues from prior prompts. No feedback was provided to the GenAI systems regarding right versus wrong answers to further mitigate AI system learning.

### Evaluation of AI outputs

Responses from each GenAI system were graded by pairs of anatomy experts blinded to the GenAI system and the timepoint at which the data were collected (Rater 1: WSB, Rater 2: ACB, Rater 3: CC, and Rater 4: ACMB; Figure 1). Raters evaluated the GenAI outputs based on two domains: (1) Factual Accuracy—the output's factual correctness relative to the query and textbook/expert responses, and (2) Superfluity—the extent of excessive/unnecessary content returned in the output as judged by anatomy experts. Superfluity evaluated whether GenAI outputs could be communicated in a reasonably succinct manner, still effective for learning.

Anatomy experts rated the quality of the outputs using a three‐point Likert scale that was piloted before implementation. Rating values were represented as “0”—the absence of the domain's desired outcomes (e.g., “inaccurate” for factual accuracy), “1”—the presence of some of the domain's desired outcomes (e.g., “partially accurate” for factual accuracy), and “2”—the complete presence of the domain's desired outcomes (e.g., “fully accurate” for factual accuracy).

To reach a consensus across all ratings, Team 2's raters served as a third party for Team 1's rating discrepancies. Each member of Team 2 independently reviewed and rated half of Team 1's discrepancies while blinded to Team 1's original ratings. The mode rating from three independent raters (e.g., rater 1 + rater 2 + rater 3/4) was used as the final consensus rating for each discrepancy. In the same way, Team 1 served as third‐party raters for Team 2's rating discrepancies. If no mode rating was achieved after the third human rating, which occurred for four items, a fourth human rater (ABW) determined the final rating.

At the project's outset, two additional scoring domains were considered: completeness and coherency. Completeness represented how well an output fully addressed the spirit of the query. Coherency represented an output's clarity and logical flow relative to the query's context. However, the absence of rater variance across these domains during rubric piloting (i.e., 100% agreement; Fleiss' kappa could not be computed as all ratings were identical across the raters) and the previously reported high quality of the GenAI systems' outputs related to these domains,^2^, ^13^, ^14^ led the research team to exclude completeness and coherency to favor a parsimonious rubric focused on factual accuracy and superfluity.

### ChatGPT o1‐preview as an AI rater

OpenAI's ChatGPT o1‐preview model with enhanced reasoning capabilities was used as an AI rater, and its rating scores were compared directly to those of the anatomy experts. The goal of using ChatGPT o1‐preview as a fifth rater was to pilot its ability to make quality rating judgments within the context of a predefined scoring rubric. If successful, AI‐assisted ratings could become an additional tool in an educational researcher's arsenal for conducting rudimentary scoring tasks. For this procedure, on September 30 and October 8, 2024, ChatGPT o1‐preview was instructed to evaluate the accuracy of the AI‐generated outputs (described above; Figure 1) compared to predetermined expert/textbook answers. Using a consistent base prompt with the rating rubric embedded into it, ChatGPT o1‐preview was given an initial question, an AI‐generated response, and the expert/textbook answers for each query. All instructions were kept identical across queries (Appendix S2). Each query was conducted in a new chat session to prevent carry‐over between sessions.

### Statistical analysis

Data were organized in Microsoft Excel (version 1906, Microsoft Corporation, Redmond, WA, USA) and analyzed using SPSS (version 29, IBM Corporation, Armonk, NY, USA), R statistical software (version 4.5.0, R Foundation for Statistical Computing, Vienna, Austria), and Stata (version 17, StataCorp, College Station, TX, USA). Descriptive statistics were used to summarize the final expert ratings across all prompts for each GenAI system. The proportion of accurate responses was computed to determine each GenAI system's factual accuracy. Percentage rating scores were computed as the mean score (*μ*_rating_) divided by 2.0 (i.e., the highest possible score) times 100. For the “superfluity” domain, an additional character count analysis was performed between GenAI systems to discern whether one system was more succinct than the other.

#### Inter‐rater agreement

Percent agreement^15^ and Cohen's kappa were used to assess inter‐rater agreement between pairs of anatomy experts and human versus AI rater pairings. Fleiss' kappa evaluated the extent of inter‐rater agreement between all three raters (e.g., ChatGPT o1‐preview + Rater 1 + Rater 2). While Cohen's kappa is widely used, it is sensitive to prevalence and bias in marginal distributions, which may distort the agreement in cases of unbalanced data.^16^ Alternatively, Gwet's AC uses an expected disagreement rate^17^ and has been shown to provide more stable estimates under such conditions.^17^ Given the variability in GenAI outputs and the differing difficulty levels across anatomical images, using both statistics allowed for a more comprehensive assessment of agreement.^18^ Cohen's kappa and Fleiss' kappa were interpreted according to Landis and Koch's recommendations for inter‐rater agreement: poor agreement (<0.00), slight agreement (0.00–0.20), fair agreement (0.21–0.40), moderate agreement (0.41–0.60), substantial agreement (0.61–0.80), and almost perfect agreement (0.81–1.00).^19^

#### Hierarchical linear modeling (HLM)

HLM is a type of multilevel modeling well‐suited for analyzing nested data structures, where individual observations (Level 1) are nested within higher‐level units (Level 2).^20^ To assess the association of both “Factual Accuracy Consensus Ratings” and “Superfluity Consensus Ratings” with the GenAI systems and predictors (timepoint and character count), mixed‐effects hierarchical linear regression models were fitted using restricted maximum likelihood estimation (REML) within Stata. The analysis followed a systematic approach, beginning with a null model to partition the total variance into within‐group and between‐group components. The final models included fixed effects for GenAI systems, timepoint, and character count, along with random intercepts for two hierarchical grouping variables: (1) “Question Prompts,” representing three question levels (Level 1), and (2) “Samples” (A, B, and C), reflecting anatomy content areas and hinged versus unhinged items (Level 2). These grouping variables accounted for clustering in the data. All assumptions for multilevel modeling were verified prior to analysis. Variance components were estimated to quantify variability attributable to Level 1 and Level 2 groupings. Intraclass correlation coefficients (ICCs) were calculated to determine the proportion of variance explained at each hierarchical level. Model fit was assessed using the log‐restricted likelihood and likelihood ratio test (LRT) to compare the mixed‐effects model with a simple linear model. Deviance statistics evaluated overall model fit, and Wald tests were used to assess the significance of individual parameters.

#### Test–retest reliability

A test–retest reliability analysis evaluated how well each GenAI system consistently returned the same answers across two different timepoints. Test–retest reliability was conducted using a two‐way mixed intraclass correlation coefficient (ICC) with average measures for “absolute agreement.” Test–retest reliability coefficients range from 0 to 1, and values were interpreted as poor (<0.50), moderate (0.51–0.75), good (0.76–0.90), and excellent (>0.900) reliability.^21^ Additionally, Krippendorff's *α* was computed as a second measure of test–retest reliability to compare the outcomes against a wider breadth of comparable literature.

To assess the influence of the time interval between GenAI outputs, model fit was compared with and without the “timepoint” variable using hierarchical linear modeling. The Akaike Information Criterion (AIC) and Bayesian Information Criterion (BIC) were used to evaluate goodness of fit.

## RESULTS

### Inter‐rater agreement between anatomy experts

Inter‐rater agreement was evaluated via percent agreement, Cohen's kappa, and Gwet's AC to ensure rating consistency was sufficient for subsequent analyses of the GenAI systems. For “factual accuracy,” irrespective of the timepoint, paired anatomy experts agreed in their ratings 82.2% to 90.0% of the time, achieving respectable Cohen's kappa values of 0.754 (substantial agreement) and 0.890 (almost perfect agreement) for each rating team, respectively. Gwet's AC values were also high (Rater 1 vs. Rater 2 = 0.806; Rater 3 vs. Rater 4 = 0.912).

The percent agreement among the paired anatomy experts for “superfluity” was 70.0% and 78.9%. The Cohen's kappa values were 0.617 (substantial agreement) and 0.639 (substantial agreement) for rating teams I and II, respectively. Gwet's AC values were respectable (Rater 1 vs. Rater 2 = 0.686; Rater 3 vs. Rater 4 = 0.764). Consensus ratings from the anatomy experts were used to analyze statistical differences between the GenAI systems, as reported below.

### AI's factual accuracy for interpreting unlabeled anatomy images

#### Model fit

The total variance explained by the multilevel model was 0.864, with Samples (A–C) and question prompts/levels accounting for 4.4% and 17.3% of the variance, respectively. While the question prompts/levels showed significant Level 1 clustering, 78.3% of the variability remained unexplained by the random effects. The Likelihood Ratio Test comparing this mixed‐effects model to a linear (univariate) model was significant (*χ* ^2^ = 10.65, *p* = 0.0049), indicating that including random effects through hierarchical modeling significantly improved the model's fit compared to conventional univariate modeling, justifying the use of multilevel modeling.

#### Major finding

**FIGURE 2:**
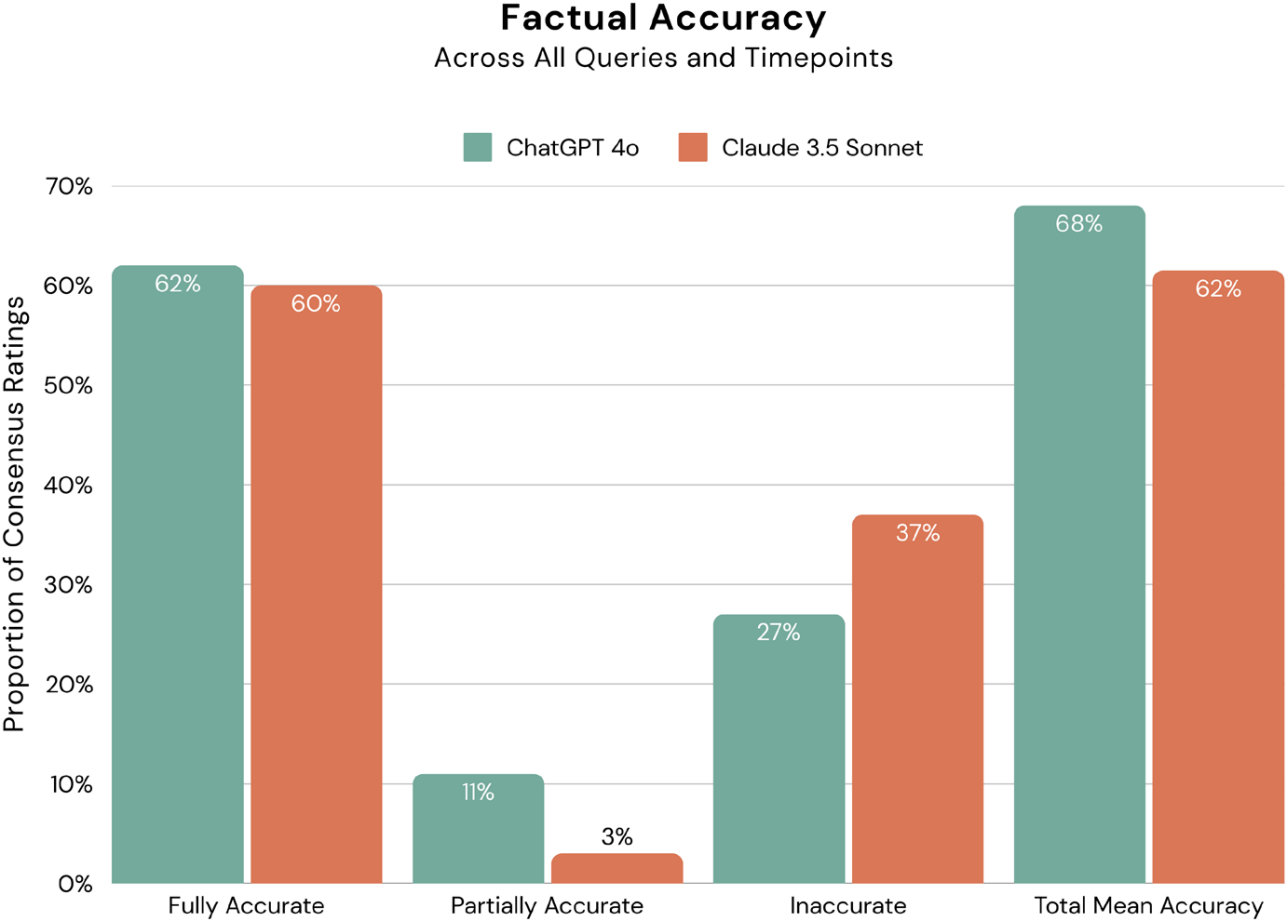
Factual accuracy outcomes of GenAI systems across all queried samples (A–C; Figure 1) and timepoints. Total mean accuracy = Average point value across 90 ratings * 100 = $\begin{array}{c} \left(\frac{\sum \;\text{of Fully}\;\left(2\;\text{points each}\right)+\text{partially accurate consensus ratings}\;\left(1\;\text{point each}\right)}{\text{Total number of ratings}\;\left(90\right)}\right) \end{array}$/2.0 (max score) * 100.

After accounting for Sample (A–C) and question prompt/level (i.e., first‐ through third‐order questioning) in the multilevel model, ChatGPT‐4o and Claude 3.5 Sonnet performed similarly (*p* = 0.319) in their abilities to accurately interpret anatomy images, and the timepoint did not affect this outcome (*p* = 0.786). On average, ChatGPT‐4o accurately identified unlabeled image‐based anatomical structures 68.0% of the time (*μ*_rating_ = 1.36) compared to Claude 3.5 Sonnet, which accurately identified structures 61.5% of the time (*μ*_rating_ = 1.23; Figure 2). These outcomes suggest that either GenAI system will answer about two of every three questions correctly when prompted to interpret an anatomy image based on first, second, or third‐order queries.

### Superfluity outcomes

#### Model fit

The total variance explained by the multilevel model was 0.246, with Samples (A–C) and question prompts/levels accounting for 9.7% and 43.1% of the variance, respectively. While the question prompts/levels showed significant clustering, 47.2% of the variability remained unexplained by the random effects. The Likelihood Ratio Test comparing this mixed‐effects model to a linear (univariate) model was significant (*χ* ^2^ = 53.05, *p* < 0.001), indicating that including random effects through hierarchical modeling significantly improved the model's fit compared to conventional univariate modeling, justifying the use of multilevel modeling.

#### Major findings

**FIGURE 3:**
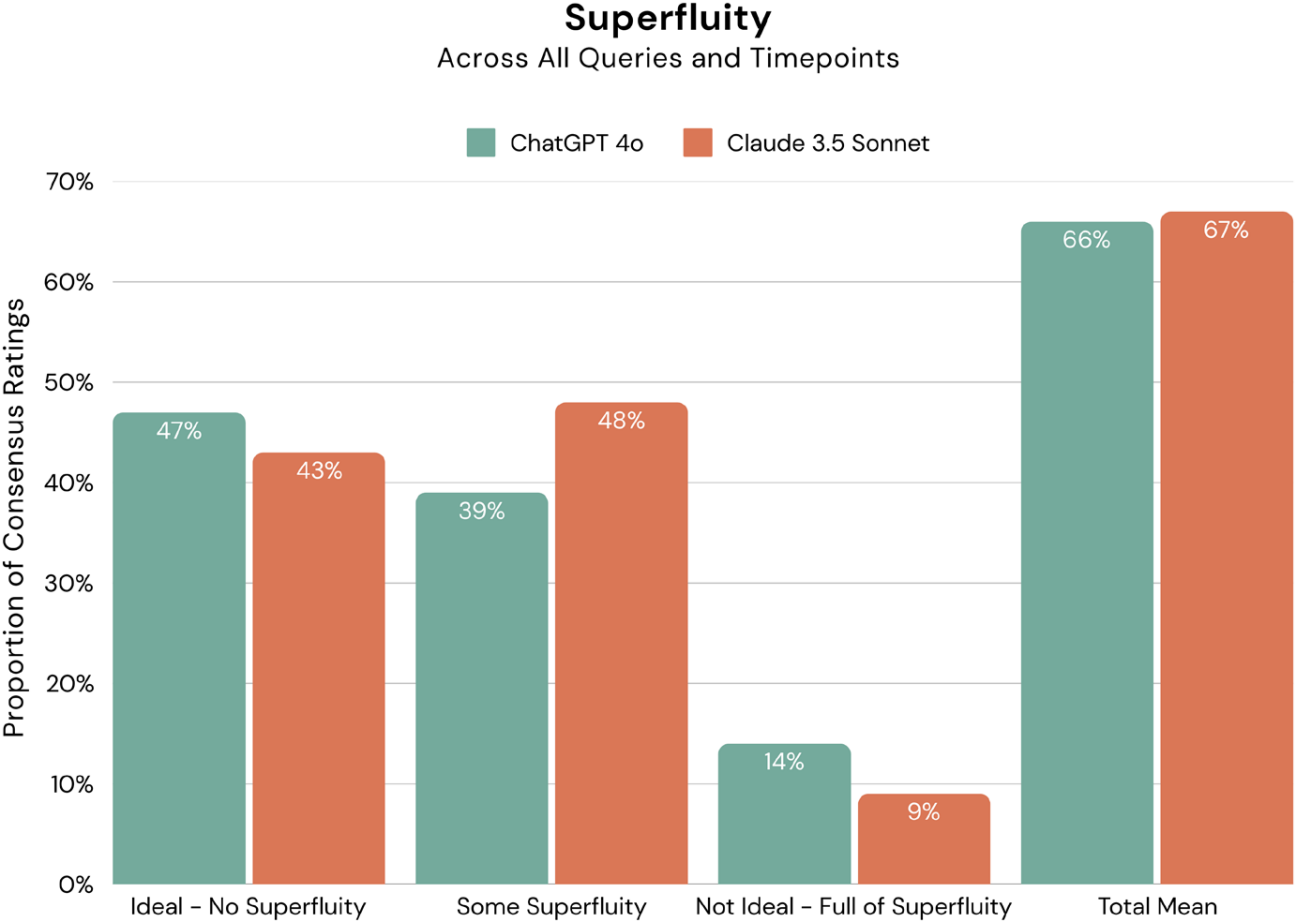
Superfluity outcomes of GenAI systems across all queried samples (A–C; Figure 1) and timepoints. Total mean accuracy = Average point value across 90 ratings * 100 = $\begin{array}{c} \left(\frac{\sum \;\text{of Fully}\;\left(2\;\text{points each}\right)+\text{partially accurate consensus ratings}\;\left(1\;\text{point each}\right)}{\text{Total number of ratings}\;\left(90\right)}\right) \end{array}$/2.0 (max score) * 100.

Holding all other variables constant in the multilevel model, Claude 3.5 Sonnet was significantly more succinct than ChatGPT‐4o (*p* = 0.012). No effect for timepoint was detected (*p* = 0.253). Claude 3.5 Sonnet averaged 189.6 (±103.4) characters per response, whereas ChatGPT‐4o averaged a character count of 237.5 (±144.2). While these outcomes suggest Claude 3.5 Sonnet was more succinct, with character counts driving the main effect, anatomy experts perceived both GenAI systems to be comparable in the succinctness of their outputs, with average rating scores of 66.0% (*μ*_rating_ = 1.32) and 67.0% (*μ*_rating_ = 1.34) for ChatGPT‐4o and Claude 3.5 Sonnet, respectively (Figure 3).

### Test–retest reliability across timepoints (T1 vs. T2) within GenAI systems

Across all prompts, ChatGPT‐4o demonstrated higher test–retest reliability (ICC = 0.770, good reliability) for factual accuracy than Claude 3.5 Sonnet (ICC = 0.505, moderate reliability). Similarly, Krippendorff's *α* values were 0.625 (moderate) and 0.338 (low) for ChatGPT‐4o and Claude 3.5 Sonnet, respectively. This suggests that ChatGPT‐4o was more consistent in returning the same factually correct answers across two different timepoints. A Wilcoxon signed‐rank test revealed no statistically significant differences (*p* ≥ 0.659) in factual accuracy outcomes between the two timepoints within either GenAI system.

Model fit was compared with and without the “timepoint” variable using hierarchical linear modeling to assess the influence of the time interval between GenAI outputs. The models excluding timepoint showed a similar fit (Factual Accuracy: AIC = 483.7; BIC 500; Superfluity: AIC = 226; BIC = 245.1) to those that included the timepoint variable (Factual Accuracy: AIC = 487.9; BIC = 507.1; Superfluity: AIC = 230.8; BIC = 253.1), suggesting no meaningful temporal effects within the short timeframe.

### ChatGPT o1‐preview as a rater versus anatomy experts

Inter‐rater agreement was evaluated via percent agreement, Cohen's/Fleiss' Kappas, and Gwet's AC to test how well ChatGPT o1‐preview performed as an AI rater compared to anatomy experts. Upon comparing ratings from each anatomy expert to those of ChatGPT o1‐preview for factual accuracy, percent agreement ranged from a low of 76.7% to a high of 87.8% (Table 1), with Cohen's kappa values ranging from 0.631 (substantial agreement) to 0.826 (almost perfect agreement; Table 1). These outcomes suggest ChatGPT o1‐preview performed reasonably well as an artificial rater and within the range of the paired anatomy experts when judging factual accuracy according to a predefined rubric with textbook answers. When judging superfluity, ChatGPT o1‐preview struggled to align with human judgments (percent agreement ≤51.1%, Cohen's kappa values ≤0.300 (slight to fair agreement); Table 1).

**TABLE 1:** Inter‐rater agreement outcomes before rating consensus was reached.

	Percent agreement	Cohen's/Fleiss' kappa (interpretation)	Gwet's AC
**Factual accuracy**			
*Timepoint 1*			
Rater 1 versus ChatGPT o1‐preview	80.0	0.651 (Substantial)	0.774
Rater 2 versus ChatGPT o1‐preview	76.7	0.631 (Substantial)	0.722
Rater 1 versus Rater 2 versus ChatGPT o1‐preview	72.2	0.678 (Substantial)	0.767
*Timepoint 2*			
Rater 3 versus ChatGPT o1‐preview	87.8	0.826 (Almost perfect)	0.867
Rater 4 versus ChatGPT o1‐preview	84.4	0.770 (Substantial)	0.815
Rater 3 versus Rater 4 versus ChatGPT o1‐preview	82.2	0.828 (Almost perfect)	0.864
**Superfluity**			
*Timepoint 1*			
Rater 1 versus ChatGPT o1‐preview	45.6	0.126 (Slight)	0.537
Rater 2 versus ChatGPT o1‐preview	51.1	0.192 (Slight)	0.585
Rater 1 versus Rater 2 versus ChatGPT o1‐preview	33.3	0.300 (Fair)	0.582
*Timepoint 2*			
Rater 3 versus ChatGPT o1‐preview	43.3	0.144 (Slight)	0.454
Rater 4 versus ChatGPT o1‐preview	42.2	0.066 (Slight)	0.461
Rater 3 versus Rater 4 versus ChatGPT o1‐preview	33.3	0.246 (Fair)	0.544

## DISCUSSION

AI technologies have shown promising potential in clinical medicine, with early use cases interpreting radiographic findings and disease states in pathology tissue samples to accelerate processing times and increase diagnostic accuracy.^22^ Recent advancements have also demonstrated AI's growing competence in medical knowledge by passing medical examinations such as the United States Medical Licensing Examination (USMLE) and specialty board examinations.^23^ Over the past 2 years of the AI arms race, AI systems have matured in their ability to provide quality and empathetic answers to patient questions,^24^ transcribe patient visits into clinical notes,^25^, ^26^ and diagnose medical conditions.^27^, ^28^, ^29^ However, AI's integration into everyday medical education practice remains in its infancy. The integration of GenAI into medical education is particularly intriguing, given its potential to offer personalized educational experiences, automated scoring and documentation, and admissions support, among other possibilities. The present study evaluated GenAI's potential within anatomy education by testing whether the features of commercially available GenAI systems were of high enough quality to recommend as a reliable resource for novice anatomy learners.

### AI's factual accuracy for interpreting unlabeled anatomy images

The present study tested GenAI's ability to interpret unlabeled anatomy images across multiple image‐based media. On average, both GenAI systems answered about two of every three questions correctly, irrespective of the content area, image‐based media, hinged versus unhinged items, or first‐, second‐, or third‐order queries. By comparison, GenAI systems designed to interpret medical imaging or to diagnose pathologies have demonstrated higher diagnostic accuracy. Across several meta‐analyses, various AI systems have demonstrated consistently high diagnostic accuracy (sensitivity ≥84% and specificity ≥84.4%) across diverse medical imaging tasks, including AI‐assisted radiography, detecting colorectal polyps, and detecting early Barrett's neoplasia.^30^, ^31^, ^32^, ^33^ Collectively, these meta‐analyses suggest that with targeted, robust training, AI can interpret medical images and return reliable and accurate information with high, human‐level diagnostic accuracy. Despite AI's low factual accuracy for interpreting unlabeled anatomy images (62%–68%), with additional training, AI may be able to enhance its anatomy image interpretation abilities to a level similar to that observed in clinical medicine applications.

Within anatomy education, ChatGPT‐3.5 answered only 33.3% (6 of 18) of text‐based anatomy queries correctly.^2^ Given the advancements in GenAI models since the release of ChatGPT‐3.5 (11/30/2022), it is unsurprising that our factual accuracy observations were higher (62%–68%) than Arun et al.'s (2024) findings. Comparatively, the customized chatbot Anatbuddy demonstrated a higher factual accuracy of 77.8%.^2^ Like the clinical diagnostic examples above, the Anatbuddy pilot work also suggests that AI could become a useful and robust tool for anatomy educators and students alike, with the right training datasets and custom knowledge bases.

### AI's superfluity

While expert ratings of superfluity accounted for both output length and the inclusion of relevant content, character count measured output length alone. This distinction is important when interpreting superfluity results, as Claude 3.5 Sonnet produced significantly shorter responses than ChatGPT‐4o, yet experts perceived both systems as comparably succinct (Average expert rating scores: ChatGPT‐4o = 66.0%; Claude 3.5 Sonnet = 67.0%). This finding suggests that expert reviewers may prioritize perceived quality, relevance, and clarity of responses over character counts and marginal gains in brevity.

### AI's reliability between timepoints

The test–retest reliability of ChatGPT's outputs across multiple occasions has been investigated in various contexts and is variable. For example, within radiology, ChatGPT‐4's ability to return consistent answers to patients' questions was moderate to high, with Krippendorff's *α* values ranging from 0.57 to 0.92.^34^ In a separate study, ChatGPT had difficulty replicating responses (Krippendorff's *α* = 0.002) when answering demanding medical content questions on the European Board Examination for Gastroenterology and Hepatology.^35^ In a third related example, ChatGPT‐4 showed moderate repeatability (Krippendorff's *α* = 0.55) in generating answers to prosthodontics questions across three timepoints (morning, afternoon, and evening).^36^ Our study suggests the repeatability of ChatGPT‐4o (Krippendorff's *α* = 0.625) to provide factually accurate answers was respectable compared to prior research. Our study also demonstrated lower test–retest reliability for Claude 3.5 Sonnet (Krippendorff's *α* = 0.338). Comparable Claude 3.5 Sonnet repeatability studies within academic medicine could not be identified for comparison.

### ChatGPT o1‐preview as an AI rater

A growing research field is exploring the practical utility of AI in educational settings as it pertains to grading, detecting human errors in reasoning and inquiry‐based methods, and providing relevant feedback to learners.^8^, ^37^, ^38^, ^39^, ^40^, ^41^ The present study adds to the existing literature by exploring ChatGPT o1‐preview as an AI rater for education‐focused research designs. In the present study, ChatGPT o1‐preview performed within the range of anatomy experts when rating the factual accuracy of GenAI outputs using a rubric with textbook answers, as demonstrated by the moderate to high inter‐rater agreement metrics (Table 1). Conversely, when tasked to judge superfluity without a scoring rubric or examples of superfluous outputs, ChatGPT o1‐preview failed to align with the judgments of anatomy experts (Table 1).

Related research exploring AI judges capable of screening records for systematic reviews has demonstrated that some AI systems are accurate and comparable to human assessors in generating inclusion/exclusion judgments.^42^ Another study concluded that AI systems were often comparable to human raters when detecting errors in students' experimentation protocols and occasionally surpassed them.^41^ An education‐focused meta‐analysis of 15 studies exploring inter‐rater agreement between humans and AI for scoring English essays demonstrated a strong pooled correlation of 0.78.^43^ Collectively, the findings in the scientific literature mimic and support the present study's outcomes, whereby inter‐rater agreement values between ChatGPT o1‐preview and anatomy experts were within acceptable human rater ranges (Cohen's kappa ≥0.631, substantial agreement) for judging factual accuracy.

Woelfle and colleagues (2024) investigated the agreement between human raters and five different AI systems (including ChatGPT‐4) regarding their ability to appraise the quality of 112 studies according to PRISMA (Preferred Reporting Items for Systematic reviews and Meta‐Analyses) and AMSTAR (A MeaSurement Tool to Assess systematic Reviews) guidelines. They found that the agreement between ChatGPT‐4 and human raters was moderate (Cohen's kappa = 0.45).^44^ Although our research aims differed, our study design was a conceptual replication of Woelfle et al.'s study (i.e., both tasked AI with judging the quality of a product according to a rubric). As such, the modestly higher Cohen's kappa values (≥0.631, substantial agreement) observed in the present study may suggest that the reasoning upgrades made to the ChatGPT o1‐preview model yielded positive improvements compared to older ChatGPT models. According to the present work and the existing literature, using AI as an assistant or counterpart to human raters is conceivable, within the boundaries of certain judgment tasks, and is likely to improve as GenAI advancements mature.

### Limitations

Given the time needed to execute, peer‐review, and publish quality studies, AI's quickly evolving landscape often makes conducting large‐scale analyses of GenAI systems impractical. As such, this pilot study was intentionally limited in scope, whereby only two of five anatomical regions were tested, and within each of the tested regions (i.e., upper limb and thorax), only five image‐based modalities were used. Future research would benefit from testing different GenAI systems (e.g., Gemini 2.0 Flash, Llama 3.2‐Vision) and a wider breadth of anatomical images and structures to draw more definitive conclusions.

### Future directions

Unfortunately, a formal analysis testing AI's factual accuracy across various imaging modalities was not feasible due to insufficient statistical power per image type. As such, future research would benefit from investigating the effects of imaging modalities on AI's factual accuracy. While this study utilized commercial GenAI tools, future work could explore the benefits of locally hosted LLMs or API‐based access to cloud‐hosted models. These models could be fine‐tuned to offer greater customizations, tailored experiences for educators and learners, and protections for a wider range of sensitive medical images (e.g., human donor images).

While drafting this manuscript, OpenAI released a vision enhancement to its Advanced Voice Mode system, referred to as ChatGPT Live Vision. This tool processes and analyzes real‐time video input from a user's camera. In a demonstration for 60 minutes, a US‐based investigative journalism television series, the host, Anderson Cooper, drew rudimentary anatomical structures (e.g., heart, brain, and liver) on a chalkboard while ChatGPT's live camera prototype analyzed Cooper's drawings and provided him with real‐time feedback on the placement accuracy of the structures. This innovative technology has direct implications for medical and anatomy education and deserves rigorous scientific testing to explore its possibilities.

At the time of data collection and analysis, ChatGPT o1‐preview was the only reasoning model on the market. With the release of other reasoning models (e.g., ChatGPT o1, DeepSeek, and Alibaba Cloud), additional investigation is warranted to determine their usefulness as an assistant AI rater for educational research projects. While ChatGPT o1‐preview performed reasonably well as an AI rater using a scoring rubric, a deeper look at the discrepancies, including a focus group with the panel of anatomy experts, may reveal patterns or explanations for why artificial reasoning diverged from expert judgments.

## CONCLUSIONS

Overall, ChatGPT‐4o and Claude 3.5 Sonnet performed similarly in their abilities to accurately interpret unlabeled anatomy images, answering approximately two‐thirds of queries correctly. ChatGPT‐4o was more reliable in replicating the same factually accurate responses. While GenAI systems hold promise as anatomy tutors for interpreting unlabeled anatomical images, their current limitations and inconsistencies underscore the need for further refinement. Additional enhancements and evaluations are needed before recommending these GenAI systems to students as anatomy education resources. At present, we recommend that students and educators use more traditional, expert‐vetted anatomy resources. Secondarily, ChatGPT o1‐preview's respectable agreement with experts as an AI rater suggests it may support educational research and offer time‐saving advantages, though its applications also require further investigation.

## AUTHOR CONTRIBUTIONS

**Lord J. Hyeamang:** Conceptualization; investigation; writing – original draft; writing – review and editing; data curation. **Tejas C. Sekhar:** Conceptualization; investigation; writing – original draft; writing – review and editing; data curation. **Emily Rush:** Conceptualization; writing – original draft; writing – review and editing; software. **Amy C. Beresheim:** Data curation; writing – original draft; writing – review and editing. **Colleen M. Cheverko:** Writing – original draft; writing – review and editing; data curation. **William S. Brooks:** Writing – original draft; writing – review and editing; data curation. **Abbey C. M. Breckling:** Writing – original draft; writing – review and editing; data curation. **M. Nazmul Karim:** Formal analysis; writing – original draft; writing – review and editing. **Christopher Ferrigno:** Conceptualization; investigation; writing – original draft; writing – review and editing; supervision; methodology. **Adam B. Wilson:** Conceptualization; investigation; writing – original draft; methodology; writing – review and editing; formal analysis; supervision; project administration.

## Supporting information


Data S1.
