# Supplementary material for: AI's ability to interpret unlabeled anatomy images and supplement educational research as an AI rater
Source: Anat Sci Educ. 2025 Jul 11;18(10):1102–13. doi: 10.1002/ase.70074 (PMC12511656; doi:10.1002/ase.70074)
Supplement: Supplementary file 1 — Data S1. [file ASE-18-1102-s001.zip › ase70074-sup-0001-Supinfo1@Supplemental Appendix Link.docx]

Link to Airtable with Supplemental Appendix information regarding images and prompts.

<https://airtable.com/app3hSUNRycOPwA21/shrbsU91JhXZDLZNs>
