# Supplementary material for: AI's ability to interpret unlabeled anatomy images and supplement educational research as an AI rater
Source: Anat Sci Educ. 2025 Jul 11;18(10):1102–13. doi: 10.1002/ase.70074 (PMC12511656; doi:10.1002/ase.70074)
Supplement: Supplementary file 1 — Data S1. [file ASE-18-1102-s001.zip › Supplement 1.docx]

[https://airtable.com/app3hSUNRycOPwA21/shrbsU91JhXZDLZNs](https://urldefense.com/v3/__https:/airtable.com/app3hSUNRycOPwA21/shrbsU91JhXZDLZNs__;!!N11eV2iwtfs!oEZpMHeZAaU_0SfZtzgHVUBTUq0liedNl3Kvhy-3Y_bTPo283-uAW-kRs5UrQejivQUuiMHO$)
